# 1-[4-(Dimethyl­amino)benzyl­idene]-4-methyl­thio­semicarbazide

**DOI:** 10.1107/S1600536810018386

**Published:** 2010-05-22

**Authors:** Yu-Feng Li, Fang-Fang Jian

**Affiliations:** a Microscale Science Institute, Department of Chemistry and Chemical Engineering, Weifang University, Weifang 261061, People’s Republic of China; b Microscale Science Institute, Weifang University, Weifang 261061, People’s Republic of China

## Abstract

In the title compound, C_11_H_16_N_4_S, an intra­molecular N—H⋯N hydrogen bond generates an *S*(5) ring. In the crystal, inversion dimers linked by pairs of N—H⋯S bonds occur, generating an *R*_2_^2^(8) loop.

## Related literature

For a related structure, see: Girgis (2006 ▶).
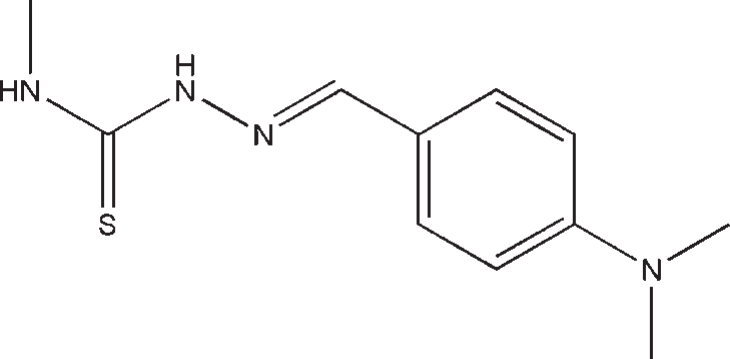


## Experimental

### 

#### Crystal data

C_11_H_16_N_4_S
*M* *_r_* = 236.34
Monoclinic, 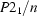

*a* = 10.517 (2) Å
*b* = 12.873 (3) Å
*c* = 10.552 (2) Å
β = 119.19 (3)°
*V* = 1247.3 (4) Å^3^
*Z* = 4
Mo *K*α radiation
μ = 0.24 mm^−1^
*T* = 293 K
0.22 × 0.20 × 0.18 mm

#### Data collection

Bruker SMART CCD diffractometer
11842 measured reflections
2847 independent reflections
2391 reflections with *I* > 2σ(*I*)
*R*_int_ = 0.031

#### Refinement

*R*[*F* ^2^ > 2σ(*F* ^2^)] = 0.055
*wR*(*F* ^2^) = 0.178
*S* = 1.12
2847 reflections
145 parameters
H-atom parameters constrained
Δρ_max_ = 0.43 e Å^−3^
Δρ_min_ = −0.26 e Å^−3^

### 

Data collection: *SMART* (Bruker 1997 ▶); cell refinement: *SAINT* (Bruker 1997 ▶); data reduction: *SAINT*; program(s) used to solve structure: *SHELXS97* (Sheldrick, 2008 ▶); program(s) used to refine structure: *SHELXL97* (Sheldrick, 2008 ▶); molecular graphics: *SHELXTL* (Sheldrick, 2008 ▶); software used to prepare material for publication: *SHELXTL*.

## Supplementary Material

Crystal structure: contains datablocks global, I. DOI: 10.1107/S1600536810018386/hb5446sup1.cif

Structure factors: contains datablocks I. DOI: 10.1107/S1600536810018386/hb5446Isup2.hkl

Additional supplementary materials:  crystallographic information; 3D view; checkCIF report

## Figures and Tables

**Table 1:** Hydrogen-bond geometry (Å, °)

*D*—H⋯*A*	*D*—H	H⋯*A*	*D*⋯*A*	*D*—H⋯*A*
N4—H4*A*⋯N2	0.86	2.22	2.613 (2)	108
N3—H3*A*⋯S1^i^	0.86	2.59	3.3890 (16)	155

Symmetry code: (i) 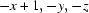.

**Fig. 1.**
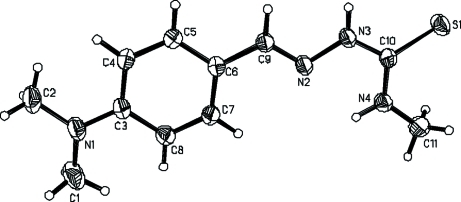
The structure of (I) showing 30% probability displacement ellipsoids.

## supplementary crystallographic information

### Experimental

A mixture of 4-(dimethylamino)benzaldehyde (0.10 mol), and 4-methylthiosemicarbazide (0.10 mol) was stirred in refluxing ethanol (10 ml) for 4 h to afford the title compound (0.083 mol, yield 83%). Colourless blocks of (I) were obtained by recrystallization from ethanol at room temperature.

### Refinement

H atoms were fixed geometrically and allowed to ride on their attached atoms, with C—H distances = 0.93-0.97 Å; N—H = 0.86Å and with *U*_iso_(H) = 1.2*U*_eq_(C,*N*) or 1.5*U*_eq_(C_methyl_).

### Crystal data

**Table:**

C_11_H_16_N_4_S	*F*(000) = 504
*M**_r_* = 236.34	*D*_x_ = 1.259 Mg m^−^^3^
Monoclinic, *P*2_1_/*n*	Mo *K*α radiation, λ = 0.71073 Å
Hall symbol: -P 2yn	Cell parameters from 2391 reflections
*a* = 10.517 (2) Å	θ = 3.2–27.5°
*b* = 12.873 (3) Å	µ = 0.24 mm^−^^1^
*c* = 10.552 (2) Å	*T* = 293 K
β = 119.19 (3)°	Block, colorless
*V* = 1247.3 (4) Å^3^	0.22 × 0.20 × 0.18 mm
*Z* = 4	

### Data collection

**Table:**

Bruker SMART CCD diffractometer	2391 reflections with *I* > 2σ(*I*)
Radiation source: fine-focus sealed tube	*R*_int_ = 0.031
graphite	θ_max_ = 27.5°, θ_min_ = 3.2°
phi and ω scans	*h* = −13→11
11842 measured reflections	*k* = −15→16
2847 independent reflections	*l* = −13→13

### Refinement

**Table:**

Refinement on *F*^2^	Primary atom site location: structure-invariant direct methods
Least-squares matrix: full	Secondary atom site location: difference Fourier map
*R*[*F*^2^ > 2σ(*F*^2^)] = 0.055	Hydrogen site location: inferred from neighbouring sites
*wR*(*F*^2^) = 0.178	H-atom parameters constrained
*S* = 1.12	*w* = 1/[σ^2^(*F*_o_^2^) + (0.1186*P*)^2^ + 0.111*P*] where *P* = (*F*_o_^2^ + 2*F*_c_^2^)/3
2847 reflections	(Δ/σ)_max_ < 0.001
145 parameters	Δρ_max_ = 0.43 e Å^−^^3^
0 restraints	Δρ_min_ = −0.26 e Å^−^^3^

### Special details

**Table:**

Geometry. All esds (except the esd in the dihedral angle between two l.s. planes) are estimated using the full covariance matrix. The cell esds are taken into account individually in the estimation of esds in distances, angles and torsion angles; correlations between esds in cell parameters are only used when they are defined by crystal symmetry. An approximate (isotropic) treatment of cell esds is used for estimating esds involving l.s. planes.
Refinement. Refinement of *F*^2^ against ALL reflections. The weighted *R*-factor wR and goodness of fit *S* are based on *F*^2^, conventional *R*-factors *R* are based on *F*, with *F* set to zero for negative *F*^2^. The threshold expression of *F*^2^ > σ(*F*^2^) is used only for calculating *R*-factors(gt) etc. and is not relevant to the choice of reflections for refinement. *R*-factors based on *F*^2^ are statistically about twice as large as those based on *F*, and *R*- factors based on ALL data will be even larger.

### Fractional atomic coordinates and isotropic or equivalent isotropic displacement parameters (Å^2^)

**Table:**

	*x*	*y*	*z*	*U*_iso_*/*U*_eq_	
S1	0.60342 (4)	0.15360 (4)	0.05021 (6)	0.0595 (2)	
N2	0.17952 (14)	0.12707 (11)	−0.13146 (16)	0.0468 (3)	
N3	0.32723 (14)	0.10421 (10)	−0.05970 (16)	0.0491 (4)	
H3A	0.3569	0.0426	−0.0272	0.059*	
C6	−0.06129 (17)	0.06639 (12)	−0.20701 (17)	0.0435 (4)	
N4	0.36809 (16)	0.27166 (11)	−0.09695 (18)	0.0537 (4)	
H4A	0.2749	0.2772	−0.1463	0.064*	
C3	−0.36590 (17)	0.09210 (14)	−0.32393 (17)	0.0481 (4)	
C7	−0.13111 (19)	0.15789 (12)	−0.2769 (2)	0.0495 (4)	
H7A	−0.0763	0.2116	−0.2848	0.059*	
C10	0.42361 (17)	0.17963 (13)	−0.04141 (17)	0.0433 (4)	
C9	0.09536 (17)	0.05248 (12)	−0.13907 (18)	0.0448 (4)	
H9A	0.1355	−0.0119	−0.1002	0.054*	
C5	−0.14729 (19)	−0.01233 (13)	−0.1990 (2)	0.0501 (4)	
H5A	−0.1036	−0.0746	−0.1543	0.060*	
C8	−0.27852 (19)	0.17078 (14)	−0.3344 (2)	0.0537 (4)	
H8A	−0.3215	0.2327	−0.3812	0.064*	
C4	−0.29596 (19)	−0.00089 (14)	−0.2553 (2)	0.0529 (4)	
H4B	−0.3502	−0.0552	−0.2478	0.063*	
N1	−0.51338 (16)	0.10490 (14)	−0.38125 (18)	0.0634 (4)	
C2	−0.5998 (2)	0.0282 (2)	−0.3574 (3)	0.0758 (6)	
H2B	−0.5423	−0.0329	−0.3152	0.114*	
H2C	−0.6834	0.0107	−0.4484	0.114*	
H2D	−0.6313	0.0560	−0.2929	0.114*	
C11	0.4544 (2)	0.36333 (16)	−0.0797 (3)	0.0730 (6)	
H12A	0.3912	0.4207	−0.1293	0.109*	
H12B	0.5174	0.3507	−0.1201	0.109*	
H12C	0.5120	0.3795	0.0216	0.109*	
C1	−0.5822 (2)	0.2016 (2)	−0.4442 (3)	0.0929 (8)	
H1A	−0.5142	0.2460	−0.4541	0.139*	
H1B	−0.6138	0.2345	−0.3828	0.139*	
H1C	−0.6647	0.1893	−0.5381	0.139*	

### Atomic displacement parameters (Å^2^)

**Table:**

	*U*^11^	*U*^22^	*U*^33^	*U*^12^	*U*^13^	*U*^23^
S1	0.0340 (3)	0.0526 (3)	0.0744 (4)	−0.00233 (15)	0.0127 (2)	−0.00072 (19)
N2	0.0336 (7)	0.0484 (7)	0.0547 (8)	0.0008 (5)	0.0187 (6)	0.0001 (6)
N3	0.0335 (6)	0.0439 (7)	0.0637 (8)	0.0010 (5)	0.0188 (6)	0.0032 (6)
C6	0.0375 (8)	0.0465 (8)	0.0464 (8)	−0.0018 (6)	0.0205 (6)	−0.0026 (6)
N4	0.0395 (7)	0.0477 (8)	0.0626 (9)	−0.0017 (5)	0.0160 (6)	0.0071 (6)
C3	0.0382 (8)	0.0593 (10)	0.0458 (8)	−0.0015 (6)	0.0197 (6)	−0.0050 (7)
C7	0.0438 (9)	0.0455 (9)	0.0594 (10)	−0.0032 (6)	0.0253 (8)	0.0021 (7)
C10	0.0382 (7)	0.0455 (8)	0.0436 (8)	−0.0027 (6)	0.0180 (6)	−0.0032 (6)
C9	0.0391 (8)	0.0437 (8)	0.0504 (8)	−0.0006 (6)	0.0210 (6)	−0.0012 (6)
C5	0.0446 (9)	0.0455 (8)	0.0572 (10)	−0.0021 (6)	0.0226 (8)	0.0055 (7)
C8	0.0447 (9)	0.0481 (9)	0.0653 (11)	0.0052 (7)	0.0245 (8)	0.0049 (7)
C4	0.0433 (9)	0.0567 (9)	0.0582 (10)	−0.0095 (7)	0.0245 (8)	0.0025 (7)
N1	0.0370 (8)	0.0805 (11)	0.0677 (10)	−0.0010 (7)	0.0218 (7)	0.0000 (8)
C2	0.0468 (10)	0.1131 (18)	0.0689 (12)	−0.0167 (11)	0.0293 (9)	−0.0044 (12)
C11	0.0597 (12)	0.0529 (10)	0.0951 (16)	−0.0073 (9)	0.0289 (11)	0.0121 (10)
C1	0.0477 (11)	0.0861 (17)	0.128 (2)	0.0166 (11)	0.0301 (13)	−0.0016 (16)

### Geometric parameters (Å, °)

**Table:**

S1—C10	1.6852 (17)	C9—H9A	0.9300
N2—C9	1.282 (2)	C5—C4	1.383 (3)
N2—N3	1.3877 (19)	C5—H5A	0.9300
N3—C10	1.348 (2)	C8—H8A	0.9300
N3—H3A	0.8600	C4—H4B	0.9300
C6—C5	1.388 (2)	N1—C1	1.431 (3)
C6—C7	1.394 (2)	N1—C2	1.444 (3)
C6—C9	1.452 (2)	C2—H2B	0.9600
N4—C10	1.325 (2)	C2—H2C	0.9600
N4—C11	1.445 (2)	C2—H2D	0.9600
N4—H4A	0.8600	C11—H12A	0.9600
C3—N1	1.372 (2)	C11—H12B	0.9600
C3—C4	1.407 (2)	C11—H12C	0.9600
C3—C8	1.408 (2)	C1—H1A	0.9600
C7—C8	1.372 (2)	C1—H1B	0.9600
C7—H7A	0.9300	C1—H1C	0.9600
			
C9—N2—N3	115.50 (14)	C7—C8—H8A	119.4
C10—N3—N2	119.27 (14)	C3—C8—H8A	119.4
C10—N3—H3A	120.4	C5—C4—C3	120.64 (15)
N2—N3—H3A	120.4	C5—C4—H4B	119.7
C5—C6—C7	117.29 (14)	C3—C4—H4B	119.7
C5—C6—C9	119.74 (14)	C3—N1—C1	121.07 (17)
C7—C6—C9	122.92 (14)	C3—N1—C2	120.89 (18)
C10—N4—C11	124.08 (16)	C1—N1—C2	117.18 (18)
C10—N4—H4A	118.0	N1—C2—H2B	109.5
C11—N4—H4A	118.0	N1—C2—H2C	109.5
N1—C3—C4	121.59 (16)	H2B—C2—H2C	109.5
N1—C3—C8	121.31 (16)	N1—C2—H2D	109.5
C4—C3—C8	117.07 (14)	H2B—C2—H2D	109.5
C8—C7—C6	121.76 (15)	H2C—C2—H2D	109.5
C8—C7—H7A	119.1	N4—C11—H12A	109.5
C6—C7—H7A	119.1	N4—C11—H12B	109.5
N4—C10—N3	116.27 (14)	H12A—C11—H12B	109.5
N4—C10—S1	124.11 (13)	N4—C11—H12C	109.5
N3—C10—S1	119.61 (13)	H12A—C11—H12C	109.5
N2—C9—C6	121.42 (15)	H12B—C11—H12C	109.5
N2—C9—H9A	119.3	N1—C1—H1A	109.5
C6—C9—H9A	119.3	N1—C1—H1B	109.5
C4—C5—C6	121.98 (15)	H1A—C1—H1B	109.5
C4—C5—H5A	119.0	N1—C1—H1C	109.5
C6—C5—H5A	119.0	H1A—C1—H1C	109.5
C7—C8—C3	121.23 (16)	H1B—C1—H1C	109.5

### Hydrogen-bond geometry (Å, °)

**Table:**

*D*—H···*A*	*D*—H	H···*A*	*D*···*A*	*D*—H···*A*
N4—H4A···N2	0.86	2.22	2.613 (2)	108
N3—H3A···S1^i^	0.86	2.59	3.3890 (16)	155

Symmetry codes: (i) −*x*+1, −*y*, −*z*.
